# Pandemic fatigue, behavioral intention and predictors of COVID-19 vaccination among individuals living with HIV in Bench Sheko Zone, in Southern Ethiopia, application of TBP: a facility based cross sectional study

**DOI:** 10.3389/fpubh.2024.1305777

**Published:** 2024-02-27

**Authors:** Melsew Setegn Alie, Gossa Fetene Abebe, Yilkal Negesse, Desalegn Girma, Amanuel Adugna

**Affiliations:** ^1^Department of Public Health, School of Public Health, College of Medicine and Health Science, Mizan-Tepi University, Mizan-Aman, Ethiopia; ^2^Department of Midwifery, College of Medicine and Health Science, Mizan-Tepi University, Mizan-Aman, Ethiopia; ^3^Department of Public Health, College of Medicine and Health Science, Debre Markos University, Debre Markos, Ethiopia

**Keywords:** COVID-19, intention, PLHIV, Southwest Ethiopia, Bench Sheko Zone

## Abstract

**Introduction:**

People living with HIV often face inequalities and negative outcomes, which make them vulnerable. To protect this population and achieve herd immunity, it is crucial for COVID-19 vaccination efforts to prioritize and encourage vaccination among people living with HIV (PLWH). However, in Ethiopia, there is a lack of motivation in this regard. To tackle this issue, a study was conducted in the Bench Sheko Zone of Southwest Ethiopia. The study aimed to assess pandemic fatigue, behavioral intention to get vaccinated, and factors influencing COVID-19 vaccine acceptance among PLWH in that region.

**Methods:**

A facility-based cross-sectional study was conducted among individuals living with HIV who were over 18 years old in Bench-Sheko Zone, located in Southwest Ethiopia. The study included a total of 590 participants from four ART healthcare facilities within the zone. The researchers utilized the Theory of Planned Behavior to examine the predictors of intention to use preconception care. Multiple linear regression analysis was employed to determine these predictors, with a *p-*value of less than 0.05 considered as indicative of a significant association. The final analysis of the study involved the use of linear regression analysis, and the measure of association was presented as the standardized B coefficient following a multivariable logistic regression analysis.

**Result:**

In the conducted study, the response rate was an impressive 98%. The researchers aimed to investigate the behavioral intention toward the COVID-19 vaccine, which was found to be 55.7%. The average age of the participants in the study was 34.65 ± 6.67. The study was the assessment of pandemic fatigue, which had a mean value of 17.22 ± 5.28. During the multivariate linear regression analysis, four predictor variables were identified. Among these, three variables, namely subjective norm, pandemic fatigue, and age, positively influenced the behavioral intention toward the COVID-19 vaccine. Comprehending these factors can assist healthcare professionals and policymakers in formulating precise interventions and strategies aimed at enhancing the acceptance and adoption of vaccines.

**Conclusion:**

The study indicates that individuals living with HIV have shown lower vaccine intention compared to previous research. The study identifies subjective norm, pandemic control measures, income, and age as predictors of individuals’ intention to receive the COVID-19 vaccine.

## Introduction

Vaccines are a crucial tool in preventing and controlling infectious diseases, saving countless lives. By vaccinating large portions of a community, herd immunity can be achieved, which effectively slows the spread of pandemics (1, 2). However, successfully controlling the COVID-19 pandemic through vaccination requires more than just vaccine efficacy and safety. It is essential that the vaccine is widely accepted by the population (3–5). COVID-19 vaccines have the potential to greatly reduce the spread of the virus from infected individuals to others, making vaccination a critical strategy in limiting the pandemic’s impact (6, 7). Unfortunately, concerns around vaccine safety, fear of contracting COVID-19, attitudes toward the vaccine, worries about genetic implications, and doubts about its effectiveness have all contributed to lower uptake of COVID-19 vaccines (8, 9). Furthermore, various beliefs and misconceptions have also influenced vaccine uptake among different population groups (10).

Vulnerable populations, such as individuals living with HIV, tend to experience worse outcomes when it comes to COVID-19 compared to those without HIV (11–13). At present, there is limited available evidence regarding the specific impact of HIV infection on the risk of experiencing adverse outcomes from COVID-19 (14). It has been observed that people living with HIV are more likely to be hospitalized and face a higher risk of mortality compared to individuals without diagnosed HIV (12, 15). For instance, a large population-based study conducted in South Africa revealed that the COVID-19 mortality risk among people living with HIV was twice as high as that of those without HIV (16). Similarly, in Spain, HIV-infected patients with COVID-19 had a higher prevalence of critical illnesses compared to those without HIV (17). Additionally, in the UK, individuals with HIV had a greater risk of COVID-19-related death compared to those without HIV (18).

Numerous studies have examined the public’s inclination to receive the COVID-19 vaccine, revealing interesting findings from different regions. In Europe, a study encompassing participants from Denmark, France, Germany, Italy, Portugal, the Netherlands, and the UK indicated a relatively high response rate of 74% (3). When exploring the factors associated with the willingness to be vaccinated against COVID-19, we can classify them into demographic and health-related predictors, as well as predictors based on theoretical behavior models. Recent studies have demonstrated that a significantly higher proportion of men (77.9%) compared to women (70.1%), particularly among men above the age of 55, exhibited a willingness to get vaccinated (3). These findings shed light on the importance of considering gender and age as potential influencing factors in vaccination intentions. It is worth noting that these statistics represent the response rates at the time of the respective studies and may have evolved since then. Nonetheless, understanding the factors that contribute to individuals’ willingness to receive the COVID-19 vaccine is crucial for public health initiatives and targeted interventions.

In other studies the intention to receive COVID-19 vaccine was 71.3% in Foch hospital France (19), 70.7% in British Columbia, Canada (20), 37.2% in Hong Hong, Chana (21), 36.4% in Jordan (22), 30.50% in Gaza strip (23), 34.2% in Gonder Ethiopia (24), 31.4% in study conducted by Belsti et al. (25), 64.9% in Ethiopia (26), 48.4% in southwestern Ethiopia (27), and 33.7% in Southwest Ethiopia (28).

The COVID-19 pandemic has had a profound impact on people’s lifestyles and well-being. As the crisis has persisted, it is not surprising that many are experiencing pandemic fatigue, which is a natural response to prolonged public health emergencies (29). However, this fatigue can lead to doubts about the effectiveness of COVID-19 mitigation strategies, which can make individuals reluctant to take necessary actions such as getting vaccinated. This can hinder efforts to end the pandemic (30, 31). Studies have shown that fear of vaccine side effects is the primary reason for vaccine hesitancy, which is a significant obstacle to achieving widespread vaccination. Pandemic fatigue and vaccine hesitancy are the most challenging issues currently facing public health officials, and they have the potential to worsen the COVID-19 situation (31–36).

Similarly, individuals who perceive themselves to be at risk for the disease (37) and those who have been advised by their healthcare provider to get vaccinated against COVID-19 (38) are more likely to express their willingness to receive a COVID-19 vaccination. Although there have been relatively few studies specifically investigating the willingness to receive a COVID-19 vaccine, numerous studies have explored the acceptance of the influenza vaccine. In this study, which aims to determine the willingness to receive a COVID-19 vaccine, I have incorporated some of the factors examined in the context of the influenza vaccine. The literature highlights several prominent characteristics that describe patients who intend to receive an influenza vaccine. It appears that males are more inclined to get vaccinated compared to females (39, 40), and older patients aged 65 and above show greater willingness to receive vaccination compared to younger patients (41). Additionally, patients with higher levels of education and income are more willing to get vaccinated (42, 43), as well as those with chronic health conditions and those who perceive their health to be less optimal (44).

The Theory of Planned Behavior (TPB) was developed by Icek Ajzen as an expansion of the Theory of Reasoned Action (45). Its purpose is to forecast an individual’s behavior based on their intention to receive vaccination. The TPB model takes into account various factors, including the individual’s attitude toward vaccination, subjective norms surrounding vaccination, perception of behavioral control over vaccination, and self-efficacy for vaccination. Research indicates that self-efficacy plays a crucial role in predicting intentions related to health (46, 47). Overall, the TPB provides a comprehensive framework for comprehending and predicting an individual’s intentions to engage in health-related behaviors, such as receiving vaccination, by considering factors such as attitudes, social norms, perceived control, and self-efficacy (45, 48–50).

Amidst the COVID-19 pandemic, research has shown that certain health beliefs are associated with people’s willingness to receive the vaccine. Specifically, individuals who have a greater belief in the likelihood of contracting a COVID-19 infection in the future and who recognize the severity of the illness are more inclined to accept vaccination, as per a study (5). To further investigate the relationship between COVID-19 and preventive behaviors, researchers have utilized the Theory of Planned Behavior (TPB) in numerous studies (51, 52). In order to effectively combat the ongoing COVID-19 pandemic, a comprehensive approach is required. This approach should include effective communication, education, and support to alleviate concerns about vaccine side effects and combat the fatigue associated with the prolonged pandemic. Despite the fact that approximately two years have passed since the pandemic began, the transmission of COVID-19 remains prevalent both in Ethiopia and worldwide. However, there is currently a lack of studies examining the concept of pandemic fatigue specifically within the Ethiopian population. Additionally, little research has been conducted to explore the potential relationship between pandemic fatigue, adherence to preventive measures aimed at reducing infection rates, and individuals’ intention to receive COVID-19 vaccinations. This knowledge gap highlights the need for comprehensive investigations to better understand the impact of pandemic fatigue on public health behaviors and attitudes toward vaccination in Ethiopia. By conducting such studies, valuable insights can be gained to inform targeted interventions and strategies to combat the pandemic effectively. To address this gap, this study aims to assess the intention and predictors of COVID-19 vaccination among individuals living with HIV in this region. Through this research, a better understanding of the factors that influence vaccine acceptance in this specific population can be gained.

## Materials and methods

### Study area and design

This research study was conducted in Bench-Sheko Zone, Southwest Ethiopian people region, a region located approximately 561 km from the capital city of Ethiopia, Addis Ababa, and 120 km from the capital city of South West Ethiopian People Regional State, Bonga. The total population of the zone is 625,345, with Mizan Aman serving as the capital city. The Bench Sheko Zone boasts 1 Teaching Hospital, 2 general hospitals, 26 health centers, 9 medium clinics, 122 primary clinics, 35 drug stores, 128 health posts, and 5 rural drug stores.

The study focused on people living with HIV who were receiving treatment in the 8 HIV/AIDS treatment centers located within these health facilities. For this particular study, four health facilities were selected at random, and a facility-based cross-sectional study was conducted from December 1 to May 30, 2022.

### Study participants

#### Source population

The source population consisted of individuals aged 18 and above who were living with HIV.

#### Study population

All individuals aged 18 and above living in the Bench Sheko Zone who have been randomly selected and meet the inclusion criteria, and who are also living with HIV.

#### Inclusion criteria

This study included individuals above the age of 18 who were living with HIV/AIDS and receiving care at selected health facilities in Bench Sheko Zone. Only those who had not completed their vaccinations were included in the study.

#### Exclusion criteria

Individuals living with HIV who experience severe illness and are unable to provide a response were not included in the study. Similarly, individuals living with HIV who are deaf and unable to communicate were also excluded from the study.

### Sample size determination and procedure

The sample size was determined by the following procedures.

Z α/_2_ = 1.96 standard score corresponding to 95% confidence interval,

Margin of error (d = 0.4),

Previous study intention to COVID-19 vaccination was 33.7% (28).

Sample size was calculated using the formula:

$n=\frac{{\left(Z_{1-\alpha /2}\right)}^{2*}P\left(1-P\right)}{d^{2}{\left(0.04\right)}^{2}}={\left(1.96\right)}^{2}0.337\;\left(1-0.337\right)=536$

Where *n* = sample size.

Z = the standard normal value at 95% CI is 1.96.

P = prevalence of vaccine hesitancy *p* = 0.337.

D = margin of error = 0.04.

The study initially aimed to have a sample size of 536 PLHIV individuals, taking into account a 10% non-response rate. However, to ensure that potential non-response was accounted for, the final sample size was increased to 590. The researchers randomly selected four out of the eight health facilities that provide ART services. After selecting the health facilities, the total sample size was proportionally allocated based on the size of each selected facility. To select the participants, the researchers used a simple random sampling method. They enrolled respondents at each health facility while they were waiting to receive healthcare services. Approximately 40 PLHIV individuals receive care and treatment follow-up services at the health facility on a daily basis. To conduct the sampling process, the researchers used simple random sampling, assigning random numbers to potential study participants in the waiting area of the health center on each data collection day.

### Study variables

This study focused on identifying the factors that influence people’s willingness to receive the COVID-19 vaccine. The researchers examined a range of socio-demographic factors such as age, marital status, family size, education level, religion, and income, as well as factors related to COVID-19, such as past experience with the virus and knowledge about it. They also considered factors related to the theory of planned behavior, including attitudes, subjective norms, and perceived behavioral control. By analyzing these factors, the researchers aimed to gain insights into what motivates individuals to get vaccinated against COVID-19.

### Data collection

The study utilized a structured questionnaire for data collection. Initially developed in English, the questionnaire drew from relevant literature and an elicitation study. To ensure linguistic accuracy, experienced instructors specializing in health education and behavioral science translated the questionnaire into Amharic. Subsequently, the Amharic version was re-translated back into English by senior instructors in the same field to assess translation consistency. The questionnaire underwent review by seven experts to establish face and content validity. The finalized Amharic version was used for data collection.

For the data collection process, three diploma holder nurses served as data collectors, supervised by a Bachelor of Science in public health. Both the collectors and supervisor received a comprehensive two-day training session covering the study’s objectives, data collection techniques, questionnaire content, and the importance of maintaining participant confidentiality. Prior to official data collection, a pre-test was conducted on 5% (40) of the total sample size. Based on the pre-test findings, any vague questions or unclear concepts in the questionnaire were modified to ensure clarity before administering it to the actual study participants. This rigorous process ensured the validity and reliability of the collected data.

### Quality control

The questionnaire was initially prepared in English and then translated into Amharic to maintain consistency and accuracy. To ensure the quality of the translation, it was re-translated back to English by another person. Before the actual data collection, the questionnaire underwent a pre-test conducted by data collectors who were not involved in the main data collection process. To ensure proper data collection, the data collectors and supervisors received comprehensive training on the data collection process, approach, and data quality management. The supervisors closely monitored the data collection activities to ensure clarity, completeness, and adherence to proper procedures. After the data collection phase, the principal investigator reviewed the questionnaire for clarity and completeness. To enhance data quality, a double data entry process was implemented using separate data sheets. This involved entering the collected data twice to minimize errors and ensure accuracy. Additionally, health education professionals, health facility vaccine providers, epidemiologists, and vaccine experts evaluated the content validity of the questionnaire. They conducted a thorough review to assess the necessity and relevance of each item using a 4-point Likert scale. The content validity index (CVI) was calculated for each item and construct, with a value of 0.833 or higher indicating an acceptable range. For construct validity, a reliability test was conducted, which yielded results within an acceptable range. The Cronbach’s alpha coefficient, a measure of internal consistency, was also found to be within an acceptable range (0.541–0.796). These measures ensure that the questionnaire accurately captures the intended constructs and provides reliable data. Overall, these rigorous steps were taken to ensure the consistency, accuracy, and quality of the questionnaire and the data collected through it.

### Measurements

The survey included questions that evaluated several aspects, including (1) demographic information and overall health status, (2) history of SARS-CoV-2 infection and any adverse reactions to the initial COVID-19 vaccination, (3) level of pandemic fatigue, (4) Knowledge toward the COVID-19 vaccine, and (5) intention to receive a COVID-19 vaccine. The measurement and the coding of each variables presented on Table 1.

**Table 1:** Measurement of each variables in the study.

Variables	Unit of measurement (descriptions)
Age	The study participants’ ages were measured in years and classified into four categories: 1 = 18–24 years, 2 = 25–34 years, 3 = 35–44 years, and 4 = 45 years old and above.
Sex	Sex of study participants were male and female
Residence	The residence of the study participant were rural and urban, we recoded the variables as 1 = rural and 2 = urban
Religion	The religion of the study participants were categorized in to four and coded as 1 = orthodox, 2 = protestant, 3 = muslim and 4 = others
Ethnicity	The ethnicity of the study participants were measured in five categories and coded as 1 = Bench and Sheka, 2 = Amhara, 3 = Keficho, 4 = Oromo and 5 = others
Educational status	The educational status of the individuals were measured in four categories coded as 1 = no formal education, 2 = primary education, 3 = secondary education and 4 = tertiary educations
Marital status	The marital status of the study participants were categorized as 1 = Ever married and 2 = not ever married
Occupation	The occupation status of the study participants were measured in four category which is coded as 1 = female sex worker, 2 = merchant, 3 = farmer and 4 = employee
Monthly income	The income level of the study participants were measured in Ethiopian birr and it was categorized as 1 = 1,000 and below Ethiopian birr, 2 = 1,001–5,000 Ethiopian birr and 3= > =5,001 Ethiopian birr
Listening to radio	The variable listening radio measured in tow category with 1 = yes and 2 = no
Watching of TV	The variable watching of TV was categorized as yes and no and it was coded as 1 = yes and 2 = no
Reading of news-paper/magazine	Reading of news-paper/magazine were measured in two categorical outcomes with coded value of 1 = yes and 2 = no
Ever diagnosed with chronic disease	The variable diagnosed chronic illness were measured as yes and no and coded as 1 = yes and 2 = no
Ever infected with COVID-19	The variable ever infected with COVID-19 were measured in two categorical outcome with coded 1 = yes and 2 = no
Did any single dose vaccinated	The variable single dose vaccine were measured in categorical outcome which was coded as 1 = yes and 2 = yes
Past vaccine side effect experience of family/friend	The experience of side effect of friend/family were measured in two category which is coded as 1 = yes and 2 = no
Perceived health status	The perceived health status of the individuals were measured in two category and coded as 1 = health and 2 = not healthy. The percentage of perceived health status was calculated from each responses.
Smoking	The smoking status of the study participant were measured in two categories which is coded as 1 = yes and 2 = no. The percentage of each value was calculated.
Overweight	The variable overweight were measured as categorized as yes and no which was coded as 1 = yes and 2 = no
Attitude	The study participants attitude was measured in six item Likert scale which was coded as 1 = strongly disagree, 2 = disagree, 3 = neutral, 4 = agree and 5 = strongly agree. The mean value of attitude was calculated from the Likert scale measurements.
Subjective norm	The study participants subjective norm was measured in six item Likert scale which was coded as 1 = strongly disagree, 2 = disagree, 3 = neutral, 4 = agree and 5 = strongly agree
Perceived behavior control	The study participants perceived behavioral control was measured in six item Likert scale which was coded as 1 = strongly disagree, 2 = disagree, 3 = neutral, 4 = agree and 5 = strongly agree. The mean value from Likert value of each items were calculated.
Intention	The study participants intention was measured in three item Likert scale which was coded as 1 = strongly disagree, 2 = disagree, 3 = neutral, 4 = agree and 5 = strongly agree. The mean value from three continuous Likert scale value was calculated.
Pandemic fatigue	The study participants pandemic fatigue was measured in six item Likert scale which was coded as 1 = strongly disagree, 2 = disagree, 3 = neutral, 4 = agree and 5 = strongly agree
Opinion	The study participants pandemic fatigue was measured in five item Likert scale which was coded as 1 = strongly disagree, 2 = disagree, 3 = neutral, 4 = agree and 5 = strongly agree
Knowledge on COVID-19	The variable knowledge was measured in eleven categorical items with coded value of 2 = yes and 1 = no for each items. The items of yes and no value of each items was summed and mean value was computed.
Prevention practice of COVID-19	The prevention practice of the study participants were measured in four categorical responses and coded as 1 = never, 2 = seldom, 3 = sometimes and 4 = most of the time. The mean value of the study participants were calculated from the items.

### Pandemic fatigue

Pandemic fatigue is a common and natural response to an extended period of dealing with a public health crisis. In a recent study, researchers aimed to assess whether individuals were experiencing demotivation and exhaustion as a result of the challenges posed by the COVID-19 pandemic. It is important to note that there is currently no established method specifically designed to measure pandemic fatigue related to COVID-19. However, for the purposes of this study, the researchers adapted a scale from a previous study. The scale consisted of six items, and participants were asked to rate their level of agreement on a Likert-type scale ranging from 1 (strongly disagree) to 5 (strongly agree). The total score on this scale can range from 6 to 42, with higher scores indicating a higher level of pandemic fatigue. The researchers categorized the pandemic fatigue scores as follows: a score of 6–22 indicated low pandemic fatigue, while a score of 23–42 indicated high pandemic fatigue (53).

### Practice of recommended measures against COVID-19 infection

The study evaluated effective measures for preventing COVID-19 infection using a specific set of 10 items. These items covered a range of preventive measures, such as wearing face masks, practicing hand hygiene, maintaining social distancing, and avoiding crowded places. Participants were asked to rate their current adherence to these measures on a 4-point Likert-type scale, ranging from 0 (never) to 3 (most of the time). The total score ranged from 0 to 30, with higher scores indicating a stronger commitment to preventive practices. To classify participants’ preventive health behavior toward COVID-19, the mean score of the items was used. A higher mean score indicated good adherence to preventive measures, while a lower mean score indicated poor adherence. For further analysis, the preventive measures score was categorized into two groups: scores of 0–23 and scores of 24–30 (24).

#### Knowledge about COVID-19 vaccine

The knowledge about the COVID-19 vaccine was assessed using eleven items, each with two response categories: 1 for “yes” and 2 for “no.” A correct answer was assigned a code of 1, while incorrect and unknown answers were coded as zero. The total score, obtained by summing the individual item scores, indicated the level of knowledge about the COVID-19 vaccine. Additionally, the mean score of the items was used to categorize the knowledge as either good or poor. If the score was at or above the mean, it was considered as good knowledge, while scores below the mean were categorized as poor knowledge (24).

#### Intention

The readiness of an individual to receive the COVID-19 vaccine can be assessed using three items, each rated on a five-point Likert scale. A higher total score indicates a stronger intention to receive the COVID-19 vaccine, with a range of scores from 3 to 15. The score of COVID-19 vaccine receive intention was obtained from the mean score of 3 related questions.

#### Attitude (AT)

The measurement was conducted using a questionnaire consisting of six items. Each item was rated on a five-point Likert scale, ranging from “strongly disagree” to “strongly agree.” The composite score, which ranged from six to thirty, reflected the individual’s overall attitude toward the COVID-19 vaccine. A higher score indicated a more positive attitude toward the vaccine.

#### Subjective norm (SN)

A study was conducted to evaluate how people perceive the level of societal approval or disapproval toward the COVID-19 vaccine. The assessment was based on four items, which were rated on a five-point Likert scale. The resulting composite score, which ranged from four to twenty, was used to determine the extent of social influence toward the COVID-19 vaccine. A higher score indicated a greater level of societal support for the vaccine.

#### Perceived behavioral control (PBC)

The research evaluated how individuals perceive their ability to influence factors related to the COVID-19 vaccine. This assessment was conducted using four items, where participants rated each item on a five-point Likert scale. The composite score ranged from four to twenty, with higher scores indicating a stronger belief in individuals’ capacity to control these factors.

### Data processing and analysis

The data collected underwent a rigorous cleaning process and was then entered into Epi-data version 4.6. Subsequently, it was exported to SPSS version 25 for analysis. During the data cleaning process, various steps were taken, including recoding variables and checking for missing values.

Based on the findings, a simple linear regression was conducted to examine the association between each independent variable and the intention to receive the COVID-19 vaccine. Only variables with a *p*-value less than 0.25 in the simple linear regression analysis were selected for inclusion in the subsequent multiple linear regression.

To ensure the validity of the analysis, several assumptions were thoroughly checked. The linearity assumptions were assessed using scatter plots for simple linear regression, and for multiple linear regression, standardized residuals were plotted against predicted values. The assumption of homoscedasticity was tested using Cameron & Trivedi’s decomposition of the IM-test with the number of predictors, which yielded insignificant results. Multicollinearity assumptions were evaluated using the variance inflation factor (VIF), and all variables demonstrated values below five, indicating no significant multicollinearity. To determine the extent to which the independent variables explain the variation in the intention to receive the COVID-19 vaccine, the R-square value was calculated. *p*-value <0.05 was considered to indicate significant association. The statistical association of dependent variables with independent variables were determined by *p-*value ≤ 0.05 (54, 55) and Standardized β-coefficient with 95% confidence interval. Additionally, the effect of each independent variable on the intention to receive the COVID-19 vaccine was interpreted using standardized β coefficients. These coefficients provide insights into the strength and direction of the relationship between the independent variables and the intention to receive the COVID-19 vaccine.

## Results

### Demographic information and overall health status

The study achieved a high response rate of 98%, indicating a strong level of participation. Among the total participants, 259 individuals (44.8%) fell into the age category of 35–44 years old. The mean age of the study participants was 34.65 ± 6.67, indicating a relatively young population. The majority of participants, 364 individuals (63.0%), were female, highlighting the gender distribution within the study. In terms of residence, 452 participants (78.2%) were from urban areas, while 279 participants (48.3%) identified as followers of the Orthodox religion, as indicated in Table 2.

**Table 2:** Demographic and health characteristics of the study participants in Bench Sheko Zone, Southwest Ethiopia.

Variables	Category	Number (Percentage)
Age Mean = 34.65, SD = 6.67	18–24 years	43 (7.4%)
	25–34 years	239 (41.3%)
	35–44 years	259 (44.8%)
	≥45 years	37 (6.4%)
Sex	Male	214 (37.0%)
	Female	364 (63.0%)
Residence	Rural	126 (21.8%)
	Urban	452 (78.2%)
Religion	Orthodox	279 (48.3%)
	Protestant	164 (28.4%)
	Muslim	113 (19.6%)
	Others^a^	22 (3.8%)
Ethnicity	Bench and sheka	222 (38.4%)
	Amhara	167 (28.9%)
	Keficho	84 (14.5%)
	Oromo	55 (9.5%)
	Other^b^	50 (8.7%)
Educational status	No formal education	104 (18.0%)
	Primary education	405 (70.1%)
	Secondary education	38 (6.6%)
	Tertiary education	31 (5.4%)
Marital status	Ever married	380 (65.7%)
	Never married	198 (34.3%)
Occupation	Female sex worker	259 (44.8%)
	Merchant	117 (20.2%)
	Farmer	107 (18.5%)
	Employee	95 (16.4%)
Estimated monthly Income	1,000 and below Ethiopian birr	53 (9.2%)
	1,001–5,000 Eth birr	324 (56.1%)
	5,001 and above	201 (34.8%)
Listening to radio	Yes	278 (48.1%)
	No	300 (51.9%)
Watching of TV	Yes	293 (50.7%)
	No	283 (49.3%)
Reading of news paper/magazine	Yes	187 (32.4%)
	No	391 (67.6%)
Ever diagnosed with chronic disease	Yes	203 (35.1%)
	No	375 (64.9%)
Ever infected with COVID-19	Yes	69 (11.9%)
	No	509 (88.1%)
Did any single dose vaccinated	Yes	45 (7.8%)
	No	533 (92.2%)
Past vaccine side effect experience of family/friend	Never	441 (76.3%)
	Mild to moderate (1–5)	92 (15.9%)
	Moderate to severe (6–10)	45 (7.8%)
Perceived health status	Not healthy	304 (52.6%)
	Healthy	274 (47.4%)
Smoking	Yes	240 (41.5%)
	No	338 (58.5%)
Overweight	Yes	236 (40.8%)
	No	342 (59.2%)

^a^Catolic, non religion, ^b^welaita, sidama.

### Pandemic fatigue

**Figure 1:**
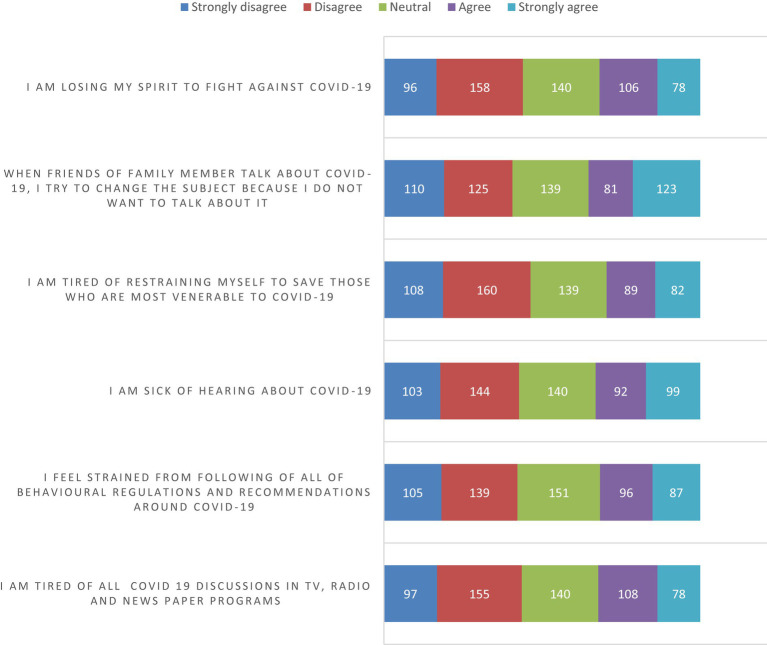
Pandemic fatigue component in the study conducted in Bench Sheko Zone, Southwest Ethiopia.

Figure 1 illustrates the distribution of agreement levels for the six items measuring pandemic fatigue. The pandemic fatigue was measured using five items such as strongly disagree, disagree, neutral, agree and strongly agree. The mean value with standard deviation was calculated from each items. The reliability of these Likert scale variables was found to be 74.9%. Among the respondents, 18.7% reported agreeing that they are tired of hearing about COVID-19, while 13.5% strongly agreed that they are exhausted from all COVID-19 discussions in the media, such as TV shows, newspapers, and radio programs. On average, the total pandemic fatigue score was 17.22 ± 5.28 (Table 3).

**Table 3:** Descriptive statistics continuous variable in the study conducted in Bench Sheko Zone, Southwest Ethiopia (*N* = 578).

Constructs	Items	Scale range	Scale mean	SD	Cronbach’s alpha
Attitude	6	6–30	16.69	5.61	0.603
Subjective norm	6	6–30	17.48	5.67	0.541
Perceived behavior control	5	5–25	14.63	4.02	0.796
Intention	3	3–15	8.77	3.00	0.669
Pandemic fatigue	6	6–30	17.22	5.28	0.749
Opinion	5	5–25	14.85	4.41	0.652

### Practice of the recommended measures against COVID-19 infection

The mean total score for prevention practices, which ranged from 0 to 36, was found to be 14.07 (SD = 3.99). Among the various preventive measures, the most commonly practiced one was the use of disinfectants, with a prevalence of 24.2% among respondents. On the other hand, a relatively low proportion of participants reported washing their hands for 20–60 s with soap (7.1%) and using hand disinfectant after touching common areas (8.3%). Avoiding dining out (13.7%) and limiting visits from or to others (8.8%) were found to be the least practiced preventive measures, as shown in Table 4.

**Table 4:** Recommended practices against COVID-19 infection in study conducted in Bench Sheko Zone, 2023.

Variables	Category			
	Never	Seldom	Some time	Most of time
Use face mask when I am at public	186 (32.2%)	213 (36.9%)	94 (16.3%)	85 (14.7%)
Avoiding crowed areas like supermarket, banks	190 (32.9%)	172 (29.8%)	126 (21.8%)	90 (15.1%)
Use hand disinfectant after touching common areas	152 (26.3%)	163 (28.2%)	216 (37.2%)	48 (8.3%)
Avoiding close contact	192 (33.2%)	147 (25.4%)	153 (26.5%)	86 (14.9%)
Avoiding large gathering, e.g., religion function, wedding or events	185 (33.2%)	176 (30.4%)	115 (19.9%)	102 (17.6%)
Avoid touching face	210 (36.3%)	120 (20.8%)	154 (26.6%)	94 (16.3%)
Using disinfectants	224 (38.8%)	129 (22.3%)	85 (14.7%)	140 (24.2%)
Staying home	218 (37.7%)	120 (20.8%)	193 (33.4%)	47 (8.1%)
Washing hand for 20–60 s with soap	228 (39.4%)	141 (24.4%)	168 (29.1%)	41 (7.1%)
I avoid dining outside	206 (35.6%)	172 (29.8%)	121 (20.9%)	79 (13.7%)
I do not go out unless it is absolutely necessary	226 (39.1%)	149 (25.8%)	97 (16.8%)	106 (18.3%)
Avoid visiting for having close friends or family member	235 (40.7%)	99 (17.1%)	193 (33.4%)	51 (8.8%)

### Knowledge on COVID-19 vaccine

The study assessed the knowledge of study participants on COVID-19 vaccine using eleven items with yes or no responses. Results showed that more than half, specifically 56.9%, of the participants were aware of the effectiveness of the COVID-19 vaccine. Additionally, 55.4% of the participants knew where to receive the vaccine. However, a significant proportion of participants, 58.0%, did not know that they were eligible to receive the vaccine, as indicated in Table 5.

**Table 5:** The component on knowledge on COVID-19 in the study conducted in Bench Sheko Zone, Southwest Ethiopia, 2023.

Variables	Category	
	Yes	No
Do you know where COVID 19 vaccine is given in Ethiopia	306 (52.9%)	272 (47.1%)
Do you know you cannot receive COVID 19 vaccine from pharmacy	275 (47.6%)	303 (52.4%)
Do you know you can receive COVID 19 vaccine	243 (42.0%)	335 (58.0%)
Do you know from where you heard about COVID 19 vaccine	320 (55.4%)	258 (44.6%)
Side effect of COVID 19 vaccine do not last more than 5 days	291 (50.3%)	287 (49.7%)
COVID 19 vaccine have mild side effect	243 (42.0%)	335 (58.0%)
COVID 19 vaccine safe for children	300 (51.9%)	278 (48.1%)
COVID 19 vaccine is safe for pregnant women	246 (42.6%)	332 (57.4%)
Do you know how may COVID 19 vaccine dose you should take	252 (43.6%)	326 (56.4%)
Do you know how long COVID 19 vaccine can be effective	329 (56.9%)	249 (43.1%)
Do you know how COVID 19 vaccine have series (1,2 or 1,2,3 booster)	290 (50.2%)	288 (49.8%)

### Opinion on COVID-19 vaccine

In this study, the opinions of individuals were evaluated using a five-item Likert scale, which ranged from 5 to 25. The mean value for this variable was calculated as 14.85, with a standard deviation of 4.41. These statistics provide insights into the central tendency and variability of participants’ responses. To assess the reliability of the items, a reliability analysis was conducted, yielding a reliability value of 65.2%. This indicates that the items used in the study demonstrate an acceptable level of internal consistency.

Analyzing the agreement level among participants, it was observed that 117 individuals (20.2% of the study participants) strongly agreed with the statement “I do not trust the COVID-19 vaccine.” This finding highlights a significant proportion of individuals who harbor concerns or doubts regarding the vaccine. Additionally, when considering the agreement level on the necessity of the COVID-19 vaccine, 130 participants (22.5%) strongly agreed that the vaccine is not necessary. This finding suggests a notable portion of individuals who question the importance or relevance of receiving the COVID-19 vaccine.

**Figure 2:**
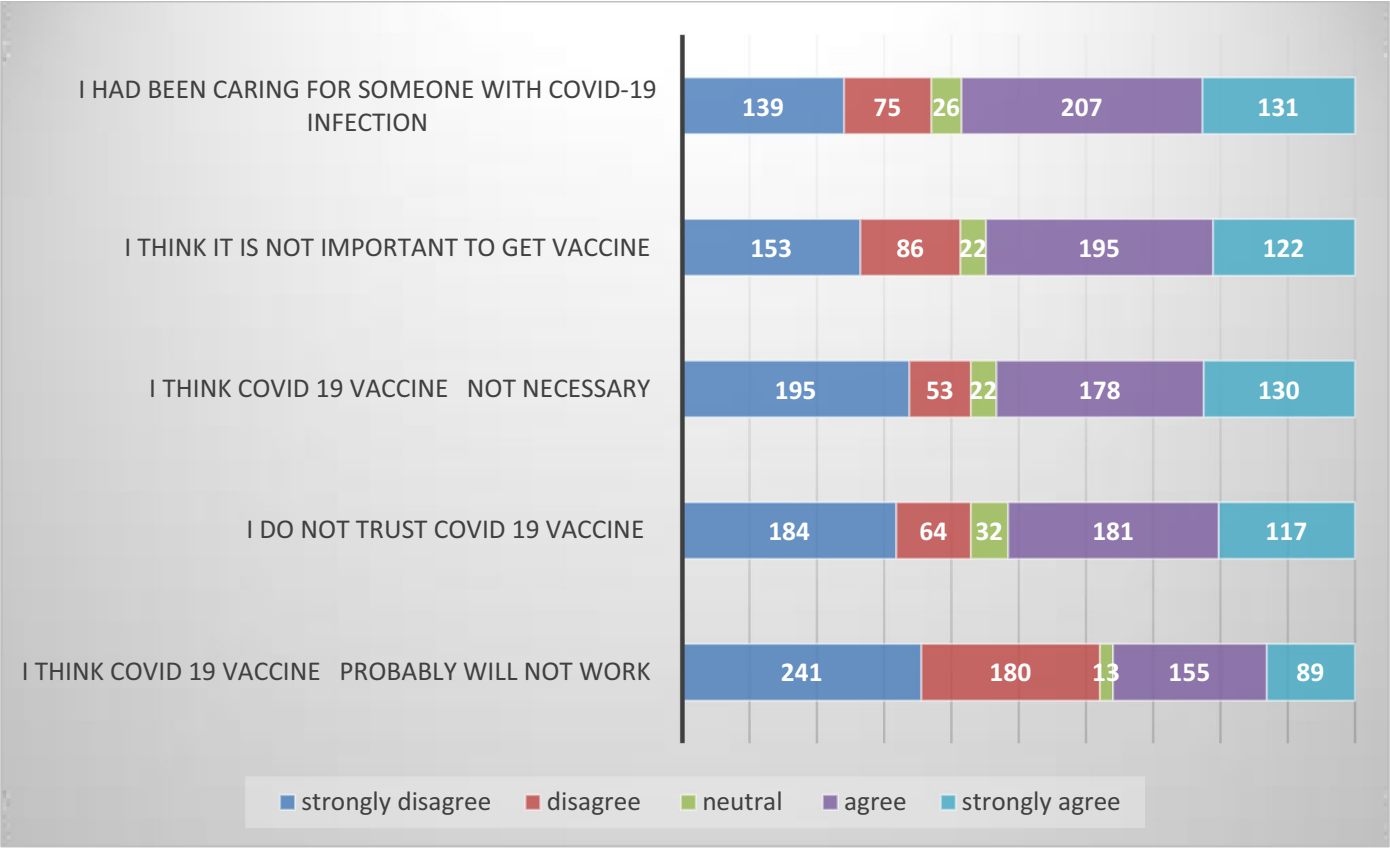
Opinion of the study participants on COVID-19 vaccine in Bench Sheko Zone, Southwest Ethiopia, 2023.

Figure 2 visually represents these findings, emphasizing the diverse range of opinions and attitudes held by the study participants toward the COVID-19 vaccine. It is crucial to consider these perspectives and address any concerns or misconceptions in order to promote vaccine acceptance and ensure the effectiveness of public health measures.

### Intention to receive COVID-19 vaccine

In a recent study, researchers used a reliable measurement scale called the Likert scale to assess participants’ intention to receive the COVID-19 vaccine. The scale consisted of three items and was found to have good internal consistency, with a high Cronbach’s alpha value of 0.669. The scores for intention to receive the vaccine were normally distributed, and the mean score was calculated to be 8.77 (Table 3). This suggests that, on average, participants expressed a positive intention to receive the vaccine. The standard deviation of 3.00 indicates that there was some variability in the intention scores within the sample. Interestingly, more than half of the participants (55.6%) scored above the mean, indicating a generally favorable intention to receive the vaccine among the study population. In fact, with a 95% confidence interval of 51.6–59.8, it was found that 55.7% of the total study participants had a higher intention to receive the COVID-19 vaccine than the mean score. Overall, these findings suggest that a significant proportion of the study population is willing to receive the COVID-19 vaccine.

### Descriptive statistics of the study

In a study, the attitude of participants was evaluated using a scale consisting of six items, with a maximum score of 30. The average attitude score was found to be 16.69, with a standard deviation of 5.61. Similarly, the mean scores for subjective norm and perceived behavioral control (PBC) were 17.48 (SD = 5.67) and 14.63 (SD = 4.02) respectively. The participants’ intention to receive the COVID-19 vaccine had an average score of 8.77, with a standard deviation of 3.0. It is noteworthy that the reliability analysis conducted for the scale variables yielded results within an acceptable range, indicating that the measurement instruments used in the study were reliable (Table 3). The health education professionals, health facility vaccine providers, epidemiologists, and vaccine experts evaluated the content validity of the questionnaire. They conducted a thorough review to assess the necessity and relevance of each item using a 4-point Likert scale. The content validity index (CVI) was calculated for each item and construct, with a value of 0.833 or higher indicating an acceptable range. For construct validity, a reliability test was conducted, which yielded results within an acceptable range. The Cronbach’s alpha coefficient, a measure of internal consistency, was also found to be within an acceptable range (0.541–0.796) (Table 3).

### Independent predictors of intention to COVID-19 vaccine

The study conducted several bivariate linear regression analyses to explore the relationship between various dependent and independent variables, including attitude, subjective norms, perceived behavioral control, opinion on the COVID-19 vaccine, pandemic fatigue, knowledge toward the COVID-19 vaccine, age, occupation, income, and reading newspaper/magazine. The analyses included Pearson’s correlation, independent sample t-test, one-way ANOVA, and bivariate linear regression.

For the multivariate linear regression analysis, the same variables were entered to determine their impact on the intention to receive the COVID-19 vaccine. The analysis identified four predictor variables that had a significant impact on the intention to receive the vaccine. The model explained 77.3% of the study variable, indicating a strong fit.

The standardized regression coefficients revealed that subjective norm had the strongest impact on the intention to receive the COVID-19 vaccine. Individuals living with HIV who believed that significant others would approve of their decision to receive the vaccine were 75.9% more likely to have the intention to receive it compared to those who did not have such perceived support. Additionally, pandemic fatigue also showed a significant influence, indicating that individuals with HIV who received continuous information from media and other sources had a 6.3% higher intention to receive the COVID-19 vaccine compared to their counterparts.

The analysis also revealed that a unit-positive change in age toward the perceived advantages of the COVID-19 vaccine increases an individual’s intention to receive it by 5.6%, while a unit-positive change in income associated with the COVID-19 vaccination showed a slight decrease in intention. Overall, these findings highlight the importance of subjective norm, pandemic fatigue, age, and income in shaping the intention to receive the COVID-19 vaccine among individuals living with HIV (Table 6).

**Table 6:** Independent predictors to behavioral intention to COVID-19 vaccinations among PLHIV, Bench Sheko Zone Southwest Ethiopia, 2023 (*n* = 578).

Variables	Unstandardized β	Standardized β	*p*-value	95% CI for β
PBC	0.034	0.46	0.43	−0.059-1.27
SN	0.402	0.759	**0.0000**	0.374–0.430
Attitude	−0.003	−0.005	0.885	−0.039-0.033
Knowledge	0.009	0.005	0.840	−0.076-0.093
Occupation	0.020	0.070	0.781	−0.120-0.160
Reading newspaper/magazine	−0.152	−0.024	0.373	−0.486-0.183
Income	−0.00006	−0.047	**0.045**	0.002–0.004
Pandemic fatigue	0.036	0.063	**0.018**	0.006–0.065
Age	0.025	0.056	**0.038**	0.001–0.049
Opinion on COVID-19 vaccine	−0.009	−0.014	0.612	−0.045-0.027

SN, direct subjective norm; PBC, perceived behavioral control.The bold values are *p*-value < 0.05.

## Discussion

This research aimed to assess the pandemic fatigue and intention of individuals who are HIV-positive to receive the COVID-19 vaccine and identifying potential factors that may influence their decision to receive COVID-19 vaccine. Based on the findings of the current study, the prevalence of individuals who have an intention to receive the COVID-19 vaccine was 55.7% (95% CI: 51.6–59.8%). Our research finding aligns with the results of vaccine intention surveys conducted in the United States, UK, and Thailand, which indicate that 57.6, 53 and 56% of the respective populations plan to get vaccinated against COVID-19 (4, 56, 57). The possible justification for this similarity can be attributed to the widespread dissemination of pandemic information and vaccine-related knowledge across the globe during the same time. Additionally, it is worth noting that both the Thailand study and our current study had a similar sex ratio, with a majority of female participants.

This finding is higher than the study finding at Jordan, 36.4% (22), Gaza strip, 30.50% (23), Hong Hong (21), Southwestern Ethiopia, 48.4% (27), in Gonder Ethiopia, 34.2% (24), and Southwest Ethiopia, 33.7% (28). One possible reason for this difference could be the variation in socio-demographic characteristics among the study participants in each study. For instance, in the study conducted in Jordan, it was observed that 54.6% of the participants belonged to the age category of 18–29 and considered themselves healthy, which led to a lower intention to vaccinate. Another possible explanation for the variation is the timing of the study means that in the current study, efforts were made to reduce exposure to vaccine misinformation and improve communication interventions. This suggests that the management of misinformation and effective communication strategies can positively influence vaccine intention and uptake.

Our research finding is lower than what is expected by WHO to achieve the 70% vaccine coverage in the community (58, 59) and in study conducted in Foch hospital French, 71.3% (19), in British Columbia, Canada, 70.7% (20), and 64.9% in Ethiopia (26). One possible explanation is that the current study included HIV positive individuals who may not have received adequate attention in the country’s campaign programs. Additionally, the study settings may have differed between the two studies, with the British Columbia study being conducted among teachers who may have had easier access to information compared to the participants in the current study.

This study also evaluated the factors that influence the intention to receive the COVID-19 vaccine among individuals living with HIV. The findings revealed that subjective norm was the only construct from the theory of planned behavior that showed a significant association in this study. Moreover, other sociodemographic variables were identified as significant factors for intention to receive COVID-19 vaccine, including income (*p* = 0.045), age (*p* = 0.038), and pandemic fatigue (*p* = 0.018). Notably, subjective norm emerged as the strongest predictor of the intention to receive the COVID-19 vaccine in this study, with a beta coefficient of 0.759 and a *p*-value of less than 0.0001. This finding is comparable with the study finding by Shmueli (34), in China (60), Norwegian population (32), Italian cancer patient (61), Southwest Ethiopia (62), and in Ethiopia (26). Encouraging significant others to present compelling evidence on the safety and efficacy of COVID-19 vaccines, while highlighting the numerous benefits of being vaccinated, can play a crucial role in influencing the intentions of the target group. By ensuring that these significant others are well-informed and confident in their knowledge, we can make a substantial impact. Additionally, either empowering patients to share their positive experiences with vaccinations, in person or through social media, can be a powerful strategy to promote vaccine acceptance.

Perceived behavioral control is one of significant predictors of intention to receive COVID-19 vaccine in previous studies (45, 48–50). While in the current study perceived behavioral control is not showed significant association with intention to vaccination. It is not unusual to observe variations in research findings regarding the Theory of Planned Behavior (TPB) and its application to people’s intention to receive vaccines. These differences can be attributed to various methodological factors, such as variances in the study population, data collection periods, and the specific measures used to assess TPB elements. Moreover, these findings suggest that the effectiveness of TPB in explaining vaccine intention is influenced by specific contexts and that there may be additional factors that moderate the relationships within TPB. Therefore, conducting further investigations into the specific conditions and circumstances surrounding vaccine intention could enhance our understanding of TPB’s explanatory power.

Attitude toward COVID-19 vaccine is not predictor for intention to receive the vaccine. It is undeniable that maintaining a positive attitude plays a crucial role in predicting one’s intention to receive the COVID-19 vaccine. However, it is equally important to reinforce this attitude by addressing and dispelling any misconceptions or misinformation surrounding the vaccines (63). Additionally, influential figures within society, including healthcare professionals, community leaders, and religious leaders, can be instrumental in promoting a positive perception of the vaccines among the public, thereby increasing their willingness to get vaccinated (64). Implementing persuasive communication strategies can also contribute to fostering a more positive attitude toward COVID-19 vaccines (65).

The study identified pandemic fatigue as the second significant predictor (*p* = 0.018) of intention to receive the COVID-19 vaccine. This finding aligns with a similar study conducted in Malaysia (53), highlighting the global concern surrounding pandemic fatigue and its impact on adherence to protective behaviors against COVID-19 (66). In this study, higher levels of pandemic fatigue, coupled with lower levels of preventive practices, were associated with decreased acceptance of vaccination. Given the potential negative effects of pandemic fatigue on adherence to preventive measures, including vaccination, it is crucial to regularly assess fatigue levels and implement appropriate interventions. To promote acceptance of preventive measures and enhance adherence, it is recommended to increase individual and social risk perception, strengthen institutional trust, and foster prosocial attitudes (66). These efforts can contribute to a stronger commitment to recommended preventive measures, including the acceptance of vaccination. The study found that when it comes to seeking COVID-19 information, the impact of message fatigue is primarily influenced by inattention rather than reactance. Message fatigue refers to the feeling of being tired or overwhelmed by constantly hearing about COVID-related information. The participants in the study reported being tired of hearing about at least one aspect of COVID-19, with mask-wearing being the most commonly mentioned topic. These findings contribute to our understanding of how message fatigue can affect people’s willingness to seek information and have important implications for designing effective public health messages.

According to a recent study, two sociodemographic factors that are significantly associated with the intention to receive the COVID-19 vaccine are age and income. The study found that participants’ age was a positive predictor of their intention to receive the vaccine, which is consistent with previous studies conducted by Luo et al. (67), Galanis et al. (68), and a study conducted in the UK (69). This may be due to the fact that as individuals age increases, their risk of morbidity and mortality from COVID-19 increases. Additionally, younger individuals may underestimate the severity of the disease and mistrust the authority responsible for approving the vaccine in their country. Another possible explanation for the higher intention to receive the vaccine among older participants is that their perceived risk of acquiring the infection may be higher. One plausible reason could be that younger individuals tend to have better overall health and fewer underlying health conditions, making them more inclined to accept and receive a COVID-19 vaccine.

The individual’s income level were the negative predictors of intention to receive COVID-19 vaccine. According to the studies conducted by Paul et al. (70) and Ruiz et al. (71) there is unusual regarding the relationship between income level and the intention to receive the COVID-19 vaccine. These studies found that refusal rates were higher among lower income individuals. However, it is important to consider the context of these findings, particularly in Ethiopia where the vaccine is distributed and administered free of charge. Therefore, a possible explanation for this finding is that lower income individuals are actually more likely to have a high intent to receive the vaccine. It is important to note that this study has a limitation in that it does not assess the intentions of individuals who are under the age of 18. It is possible that the findings from this younger population may differ from those who are 18 years or older. Additionally, it is important to acknowledge that the cross-sectional nature of this study does not allow for the determination of cause-and-effect relationships between the identified factors.

## Conclusion

The intention to receive the COVID-19 vaccine is lower than the target set by the World Health Organization and previous studies. The current study aims to explore the theory of planned behavior constructs and demographic factors that determine people’s willingness to get vaccinated, with the goal of facilitating the vaccination process. The findings indicate that two demographic factors, age and income, influence the intention to get vaccinated. Subjective norms, a key component of the Theory of Planned Behavior (TPB), were found to have a highly positive association with intention to vaccinate, while attitude and perceived behavioral control did not show a significant association with vaccine intention. Another predictor of vaccination intention is pandemic fatigue. All four factors were found to be significant predictors of the intention to receive the COVID-19 vaccine among people living with HIV. Therefore, the results of this study clearly demonstrate that the TPB is an effective model for predicting and explaining vaccine uptake intentions. Additionally, there is substantial evidence from the literature suggesting that intentions ultimately translate into actual behavior (64, 72).

The findings of this study provide valuable insights for developing a comprehensive roadmap for a successful COVID-19 vaccination program tailored by demographic and behavioral factors to the specific population. In addition to promoting vaccine intention, it is important to consider subjective norms, age of individuals and income level. A significant number of respondents indicated that their family, friends, and other important individuals in their lives would support their decision to get vaccinated. Participants also expressed confidence in their ability to receive the vaccine. This implies that social structure and social networks are very crucial for national vaccine program implementation and success. Therefore, it is essential to pay attention to the attitudes of significant others toward vaccines. Furthermore, it would be beneficial to involve individuals with lower income, those experiencing pandemic fatigue, and older age groups in promoting the COVID-19 vaccination drive. This broader engagement can expand the influence of significant individuals, thereby reinforcing subjective norms and strengthening the relationship between vaccination intention and uptake.

It is worth noting that having a positive attitude toward vaccines, strong subjective norms, and moderate perceived behavioral control are key predictors of intention to receive COVID-19 vaccines. However, to achieve optimal results, it is crucial to carefully address misinformation on social media and other platforms. COVID-19 vaccination campaigns should prioritize transparency and dispel rumors regarding the safety and efficacy of vaccines and considering the sociodemographic characteristic of individuals like age and income level. Additionally, creating an enabling environment that supports vaccine uptake is essential for boosting vaccine confidence.

## Data availability statement

The data analysed in this research will be made available upon reasonable request from the corresponding author.

## Ethics statement

The studies involving humans were approved by the study received ethical clearance from the Institutional Review Board of Mizan Tepi University, College of Health Science (Ref #0028/23). The studies were conducted in accordance with the local legislation and institutional requirements. The participants provided their written informed consent to participate in this study.

## Author contributions

MA: Conceptualization, Formal analysis, Methodology, Resources, Software, Validation, Visualization, Writing – original draft, Writing – review & editing. GA: Conceptualization, Data curation, Investigation, Methodology, Supervision, Writing – review & editing. YN: Data curation, Formal analysis, Methodology, Writing – original draft, Writing – review & editing. DG: Data curation, Resources, Supervision, Validation, Visualization, Writing – original draft, Writing – review & editing. AA: Conceptualization, Formal analysis, Funding acquisition, Investigation, Resources, Validation, Writing – review & editing.

## References

[1] Kadkhoda K. Herd immunity to COVID-19: Alluring and elusive. Oxford: Oxford University Press US. (2021). p. 471–472, 155. 10.1093/ajcp/aqaa272 PMC7929447 33399182

[2] Ella KM Mohan VK. Coronavirus vaccine: light at the end of the tunnel. Indian Pediatr. (2020) 57:407–10. doi: 10.1007/s13312-020-1812-z, PMID: 32291382 PMC7240229

[3] Sallam M. COVID-19 vaccine hesitancy worldwide: a concise systematic review of vaccine acceptance rates. Vaccines. (2021) 9:160. doi: 10.3390/vaccines9020160 33669441 PMC7920465

[4] Fisher KA Bloomstone SJ Walder J Crawford S Fouayzi H Mazor KM. Attitudes toward a potential SARS-CoV-2 vaccine: a survey of US adults. Ann Intern Med. (2020) 173:964–73. doi: 10.7326/M20-3569, PMID: 32886525 PMC7505019

[5] French J Deshpande S Evans W Obregon R. Key guidelines in developing a pre-emptive COVID-19 vaccination uptake promotion strategy. Int J Environ Res Public Health. (2020) 17:5893. doi: 10.3390/ijerph17165893, PMID: 32823775 PMC7459701

[6] Freeman D Loe BS Chadwick A Vaccari C Waite F Rosebrock L. COVID-19 vaccine hesitancy in the UK: the Oxford coronavirus explanations, attitudes, and narratives survey (oceans) II. Psychol Med. (2022) 52:3127–41. doi: 10.1017/S0033291720005188, PMID: 33305716 PMC7804077

[7] El-Elimat T AbuAlSamen MM Almomani BA Al-Sawalha NA Alali FQ. Acceptance and attitudes toward COVID-19 vaccines: a cross-sectional study from Jordan. PLoS One. (2021) 16:e0250555. doi: 10.1371/journal.pone.0250555, PMID: 33891660 PMC8064595

[8] Burke PF Masters D Massey G. Enablers and barriers to COVID-19 vaccine uptake: an international study of perceptions and intentions. Vaccine. (2021) 39:5116–28. doi: 10.1016/j.vaccine.2021.07.056, PMID: 34340856 PMC8299222

[9] Altulaihi BA Alharbi KG Alaboodi TA Alkanhal HM Alobaid MM Aldraimly MA. Factors and determinants for uptake of COVID-19 vaccine in a medical university in Riyadh, Saudi Arabia. Cureus. (2021) 13:e17768. doi: 10.7759/cureus.17768 34659979 PMC8494158

[10] Green MS Abdullah R Vered S Nitzan D. A study of ethnic, gender and educational differences in attitudes toward COVID-19 vaccines in Israel–implications for vaccination implementation policies. Isr J Health Policy Res. (2021) 10:1–12. doi: 10.1186/s13584-021-00458-w 33741063 PMC7977502

[11] Bogart LM Ojikutu BO Tyagi K Klein DJ Mutchler MG Dong L. COVID-19 related medical mistrust, health impacts, and potential vaccine hesitancy among black Americans living with HIV. J Acquir Immune Defic Syndr. (2021) 86:200–7. doi: 10.1097/QAI.0000000000002570, PMID: 33196555 PMC7808278

[12] Tesoriero JM Swain C-AE Pierce JL Zamboni L Wu M Holtgrave DR. COVID-19 outcomes among persons living with or without diagnosed HIV infection in New York state. JAMA Netw Open. (2021) 4:37069. doi: 10.1001/jamanetworkopen.2020.37069 PMC7859843 33533933

[13] Waterfield KC Shah GH Etheredge GD Ikhile O. Consequences of COVID-19 crisis for persons with HIV: the impact of social determinants of health. BMC Public Health. (2021) 21:1–7. doi: 10.1186/s12889-021-10296-9 33546659 PMC7863613

[14] Fung M Babik JM. COVID-19 in immunocompromised hosts: what we know so far. Clin Infect Dis. (2021) 72:340–50. doi: 10.1093/cid/ciaa863, PMID: 33501974 PMC7337668

[15] Sigel K Swartz T Golden E Paranjpe I Somani S Richter F. Coronavirus 2019 and people living with human immunodeficiency virus: outcomes for hospitalized patients in new York City. Clin Infect Dis. (2020) 71:2933–8. doi: 10.1093/cid/ciaa880, PMID: 32594164 PMC7337691

[16] Davies M. Risk factors for COVID-19 death in a population cohort study from the Western Cape Province. South Africa, CID, this edition, Clin Infect Dis. (2021) 73, e2005–e2015. doi: 10.1093/cid/ciaa1198 32860699 PMC7499501

[17] Vizcarra P Pérez-Elías MJ Quereda C Moreno A Vivancos MJ Dronda F. Description of COVID-19 in HIV-infected individuals: a single-Centre, prospective cohort. The Lancet HIV. (2020) 7:e554–64. doi: 10.1016/S2352-3018(20)30164-8, PMID: 32473657 PMC7255735

[18] Bhaskaran K Rentsch CT MacKenna B Schultze A Mehrkar A Bates CJ. HIV infection and COVID-19 death: a population-based cohort analysis of UK primary care data and linked national death registrations within the OpenSAFELY platform. The Lancet HIV. (2021) 8:e24–32. doi: 10.1016/S2352-3018(20)30305-2, PMID: 33316211 PMC7773630

[19] Vallée A Fourn E Majerholc C Touche P Zucman D. COVID-19 vaccine hesitancy among French people living with HIV. Vaccine. (2021) 9:302. doi: 10.3390/vaccines9040302, PMID: 33804808 PMC8063788

[20] Kaida A Brotto LA Murray MC Côté HC Albert AY Nicholson V. Intention to receive a COVID-19 vaccine by HIV status among a population-based sample of women and gender diverse individuals in British Columbia, Canada. AIDS Behav. (2022) 26:2242–55. doi: 10.1007/s10461-022-03577-w, PMID: 35020094 PMC8753016

[21] Wong MC Wong EL Huang J Cheung AW Law K Chong MK. Acceptance of the COVID-19 vaccine based on the health belief model: a population-based survey in Hong Kong. Vaccine. (2021) 39:1148–56. doi: 10.1016/j.vaccine.2020.12.083 33461834 PMC7832076

[22] Al-Qerem WA Jarab AS. COVID-19 vaccination acceptance and its associated factors among a middle eastern population. Front Public Health. (2021) 9:632914. doi: 10.3389/fpubh.2021.632914, PMID: 33643995 PMC7902782

[23] Abusalem S Abuhammad S Sha S Mar MM Aljeesh Y Eldeirawi KM. Intentions to receive COVID-19 vaccination among people in Gaza strip. Electron J Gen Med. (2022) 19:12413. doi: 10.29333/ejgm/12413

[24] Handebo SWM Shitu K Kassie A. Determinant of intention to receive COVID-19 vaccine among school teachers in Gondar City, Northwest Ethiopia. PLoS One. (2021) 16:e0253499. doi: 10.1371/journal.pone.0253499, PMID: 34166399 PMC8224841

[25] Belsti Y Gela YY Akalu Y Dagnew B Getnet M Abdu Seid M. Willingness of Ethiopian population to receive COVID-19 vaccine. J Multidiscip Healthc. (2021) 14:1233–43. doi: 10.2147/JMDH.S312637, PMID: 34093019 PMC8169050

[26] Asmare GAK Atnafu N Asnake G Yeshambel A Alem E Chekol E. Behavioral intention and its predictors toward COVID-19 vaccination among people most at risk of exposure in Ethiopia: applying the theory of planned behavior model. Hum Vaccin Immunother. (2021) 17:4838–45. doi: 10.1080/21645515.2021.2011651 35213947 PMC8903977

[27] Angelo AT Alemayehu DS Dachew AM. Health care workers intention to accept COVID-19 vaccine and associated factors in southwestern Ethiopia, 2021. PLoS One. (2021) 16:e0257109. doi: 10.1371/journal.pone.0257109, PMID: 34478470 PMC8415602

[28] Mesfin Y Argaw M Geze S Zewdu BT. Factors associated with intention to receive COVID-19 vaccine among HIV positive patients attending ART clinic in Southwest Ethiopia. Patient Prefer Adherence. (2021) 15:2731–8. doi: 10.2147/PPA.S342801, PMID: 34916783 PMC8668249

[29] World Health Organization. Pandemic fatigue–reinvigorating the public to prevent COVID-19: Policy framework for supporting pandemic prevention and management. Regional Office for Europe: World Health Organization (2020).

[30] Reicher S Drury J. Pandemic fatigue? How adherence to covid-19 regulations has been misrepresented and why it matters. BMJ. (2021):372. doi: 10.1136/bmj.n137 33461963

[31] Lindholt MF Jørgensen F Bor A Petersen MB. Public acceptance of COVID-19 vaccines: cross-national evidence on levels and individual-level predictors using observational data. BMJ Open. (2021) 11:e048172. doi: 10.1136/bmjopen-2020-048172, PMID: 34130963 PMC8210695

[32] Wolff K. COVID-19 vaccination intentions: the theory of planned behavior, optimistic bias, and anticipated regret. Front Psychol. (2021) 12:648289. doi: 10.3389/fpsyg.2021.648289, PMID: 34220620 PMC8241938

[33] Thaker J Ganchoudhuri S. The role of attitudes, norms, and efficacy on shifting COVID-19 vaccine intentions: a longitudinal study of COVID-19 vaccination intentions in New Zealand. Vaccine. (2021) 9:1132. doi: 10.3390/vaccines9101132, PMID: 34696240 PMC8570329

[34] Shmueli L. Predicting intention to receive COVID-19 vaccine among the general population using the health belief model and the theory of planned behavior model. BMC Public Health. (2021) 21:1–13. doi: 10.1186/s12889-021-10816-7 33902501 PMC8075011

[35] Zhang KC Fang Y Cao H Chen H Hu T Chen Y. Behavioral intention to receive a COVID-19 vaccination among Chinese factory workers: cross-sectional online survey. J Med Internet Res. (2021) 23:e24673. doi: 10.2196/24673, PMID: 33646966 PMC7945977

[36] Ala'a B Tarhini Z Akour A. A swaying between successive pandemic waves and pandemic fatigue: Where does Jordan stand? Amsterdam: Elsevier (2021). 102298 p. 10.1016/j.amsu.2021.102298 PMC8051171 33880181

[37] Dror AA Eisenbach N Taiber S Morozov NG Mizrachi M Zigron A. Vaccine hesitancy: the next challenge in the fight against COVID-19. Eur J Epidemiol. (2020) 35:775–9. doi: 10.1007/s10654-020-00671-y 32785815 PMC8851308

[38] Reiter PL Pennell ML Katz ML. Acceptability of a COVID-19 vaccine among adults in the United States: how many people would get vaccinated? Vaccine. (2020) 38:6500–7. doi: 10.1016/j.vaccine.2020.08.043 32863069 PMC7440153

[39] Schmid P Rauber D Betsch C Lidolt G Denker M-L. Barriers of influenza vaccination intention and behavior–a systematic review of influenza vaccine hesitancy, 2005–2016. PLoS One. (2017) 12:e0170550. doi: 10.1371/journal.pone.0170550, PMID: 28125629 PMC5268454

[40] Bish A Yardley L Nicoll A Michie S. Factors associated with uptake of vaccination against pandemic influenza: a systematic review. Vaccine. (2011) 29:6472–84. doi: 10.1016/j.vaccine.2011.06.107 21756960

[41] Velan B Kaplan G Ziv A Boyko V Lerner-Geva L. Major motives in non-acceptance of a/H1N1 flu vaccination: the weight of rational assessment. Vaccine. (2011) 29:1173–9. doi: 10.1016/j.vaccine.2010.12.006, PMID: 21167862

[42] Fabry P Gagneur A Pasquier J-C. Determinants of a (H1N1) vaccination: cross-sectional study in a population of pregnant women in Quebec. Vaccine. (2011) 29:1824–9. doi: 10.1016/j.vaccine.2010.12.109, PMID: 21219988

[43] Schwarzinger M Flicoteaux R Cortarenoda S Obadia Y Moatti J-P. Low acceptability of a/H1N1 pandemic vaccination in French adult population: did public health policy fuel public dissonance? PLoS One. (2010) 5:e10199. doi: 10.1371/journal.pone.0010199, PMID: 20421908 PMC2856629

[44] Rubin GJ Potts HW Michie S. The impact of communications about swine flu (influenza a H1N1v) on public responses to the outbreak: results from 36 national telephone surveys in the UK. Health Technol Assess. (2010) 14:183–266. doi: 10.3310/hta14340-03, PMID: 20630124

[45] Ajzen I Fishbein M. Attitude-behavior relations: a theoretical analysis and review of empirical research. Psychol Bull. (1977) 84:888–918. doi: 10.1037/0033-2909.84.5.888

[46] Liao Q Cowling BJ Lam WWT Fielding R. Factors affecting intention to receive and self-reported receipt of 2009 pandemic (H1N1) vaccine in Hong Kong: a longitudinal study. PLoS One. (2011) 6:e17713. doi: 10.1371/journal.pone.0017713, PMID: 21412418 PMC3055876

[47] McClenahan C Shevlin M Adamson G Bennett C O'Neill B. Testicular self-examination: a test of the health belief model and the theory of planned behaviour. Health Educ Res. (2007) 22:272–84. doi: 10.1093/her/cyl076, PMID: 16885203

[48] Yang ZJ. Predicting young adults’ intentions to get the H1N1 vaccine: an integrated model. J Health Commun. (2015) 20:69–79. doi: 10.1080/10810730.2014.904023, PMID: 24870976

[49] Lau JT Yeung NC Choi K Cheng MY Tsui H Griffiths S. Factors in association with acceptability of a/H1N1 vaccination during the influenza a/H1N1 pandemic phase in the Hong Kong general population. Vaccine. (2010) 28:4632–7. doi: 10.1016/j.vaccine.2010.04.076, PMID: 20457289 PMC7131323

[50] Myers L Goodwin R. Using a theoretical framework to determine adults' intention to vaccinate against pandemic swine flu in priority groups in the UK. Public Health. (2012) 126:S53–6. doi: 10.1016/j.puhe.2012.05.024, PMID: 22784583

[51] Lin CY Imani V Majd NR Ghasemi Z Griffiths MD Hamilton K. Using an integrated social cognition model to predict COVID-19 preventive behaviours. Br J Health Psychol. (2020) 25:981–1005. doi: 10.1111/bjhp.12465, PMID: 32780891 PMC7436576

[52] Thu TPB Ngoc PNH Hai NM. Effect of the social distancing measures on the spread of COVID-19 in 10 highly infected countries. Sci Total Environ. (2020) 742:140430. doi: 10.1016/j.scitotenv.2020.140430, PMID: 32623158 PMC7307990

[53] Wong LPAH Siaw YL Muslimin M Lai LL Lin Y Hu Z. Intention to receive a COVID-19 vaccine booster dose and associated factors in Malaysia. Hum Vaccin Immunother. (2022) 18:2078634. doi: 10.1080/21645515.2022.2078634, PMID: 35648441 PMC9481074

[54] Dahiru T. P-value, a true test of statistical significance? A cautionary note. Ann Ibadan Postgraduate Med. (2008) 6:21–6. doi: 10.4314/aipm.v6i1.64038 PMID: 25161440 PMC4111019

[55] Sterne JA Smith GD. Sifting the evidence—what's wrong with significance tests? Phys Ther. (2001) 81:1464–9. doi: 10.1093/ptj/81.8.1464, PMID: 28206639

[56] McKie R. COVID-19: only half of Britons would definitely have vaccination. The Guardian. (2020).

[57] Boon-Itt S Rompho N Jiarnkamolchurn S Skunkan Y. Interaction between age and health conditions in the intention to be vaccinated against COVID-19 in Thailand. Hum Vaccin Immunother. (2021) 17:4816–22. doi: 10.1080/21645515.2021.1979378, PMID: 34613887 PMC8903902

[58] World Health Organization. WHO SAGE roadmap for prioritizing uses of COVID-19 vaccines: An approach to optimize the global impact of COVID-19 vaccines, based on public health goals, global and national equity, and vaccine access and coverage scenarios, first issued 20 October 2020, updated: 13 November 2020, updated: 16 July 2021, latest update: 21 January 2022. Geneva: World Health Organization (2022).

[59] Doshi P. COVID-19: do many people have pre-existing immunity? BMJ. (2020):370. doi: 10.1136/bmj.m3563 32943427

[60] Dou K Yang J Wang L-X Li J-B. Theory of planned behavior explains males’ and females’ intention to receive COVID-19 vaccines differently. Hum Vaccin Immunother. (2022) 18:2086393. doi: 10.1080/21645515.2022.2086393, PMID: 35749588 PMC9620988

[61] Servidio R Malvaso A Vizza D Valente M Campagna MR Iacono ML. The intention to get COVID-19 vaccine and vaccine uptake among cancer patients: an extension of the theory of planned behaviour (TPB). Support Care Cancer. (2022) 30:7973–82. doi: 10.1007/s00520-022-07238-5 35752690 PMC9244196

[62] Sisay AL Getahun HA Getachew N Gebremedhin T Sebero FM Birhanu A. Barriers and intention to get vaccinated for COVID-19 and associated factors among adults in Southwest Ethiopia: a theory of planned behavior approach. Infection Drug Resist. (2023) 16:5741–54. doi: 10.2147/IDR.S419952, PMID: 37670980 PMC10476652

[63] Benis A Seidmann A Ashkenazi S. Reasons for taking the COVID-19 vaccine by US social media users. Vaccine. (2021) 9:315. doi: 10.3390/vaccines9040315, PMID: 33805283 PMC8067223

[64] Xiao X Wong RM. Vaccine hesitancy and perceived behavioral control: a meta-analysis. Vaccine. (2020) 38:5131–8. doi: 10.1016/j.vaccine.2020.04.076, PMID: 32409135

[65] W-yS C Hunt YM Beckjord EB Moser RP Hesse BW. Social media use in the United States: implications for health communication. J Med Internet Res. (2009) 11:e1249. doi: 10.2196/jmir.1249 PMC2802563 19945947

[66] Petherick A Goldszmidt R Andrade EB Furst R Hale T Pott A. A worldwide assessment of changes in adherence to COVID-19 protective behaviours and hypothesized pandemic fatigue. Nat Hum Behav. (2021) 5:1145–60. doi: 10.1038/s41562-021-01181-x, PMID: 34345009

[67] Luo C Yang Y Liu Y Zheng D Shao L Jin J. Intention to COVID-19 vaccination and associated factors among health care workers: a systematic review and meta-analysis of cross-sectional studies. Am J Infect Control. (2021) 49:1295–304. doi: 10.1016/j.ajic.2021.06.020, PMID: 34273461 PMC8278862

[68] Galanis P Vraka I Fragkou D Bilali A Kaitelidou D. Intention of healthcare workers to accept COVID-19 vaccination and related factors: a systematic review and meta-analysis. Asian Pac J Trop Med. (2021) 14:543–54. doi: 10.4103/1995-7645.332808

[69] Sherman SM Smith LE Sim J Amlôt R Cutts M Dasch H. COVID-19 vaccination intention in the UK: results from the COVID-19 vaccination acceptability study (CoVAccS), a nationally representative cross-sectional survey. Hum Vaccin Immunother. (2021) 17:1612–21. doi: 10.1080/21645515.2020.1846397, PMID: 33242386 PMC8115754

[70] Paul E Steptoe A Fancourt D. Attitudes towards vaccines and intention to vaccinate against COVID-19: implications for public health communications. The Lancet Regional Health–Europe. (2021) 1. doi: 10.1016/j.lanepe.2020.100012 PMC7834475 33954296

[71] Ruiz JB Bell RA. Predictors of intention to vaccinate against COVID-19: results of a nationwide survey. Vaccine. (2021) 39:1080–6. doi: 10.1016/j.vaccine.2021.01.010, PMID: 33461833 PMC7794597

[72] Holland T. Humour styles: Predictors of perceived stress and self-efficacy with gender and age differences. (2016). Available at: http://hdl.handle.net/10788/3182

